# Circulating Monocyte-Like Myeloid Derived Suppressor Cells and CD16 Positive Monocytes Correlate With Immunological Responsiveness of Tuberculosis Patients

**DOI:** 10.3389/fcimb.2022.841741

**Published:** 2022-03-14

**Authors:** Nicolás O. Amiano, Joaquín M. Pellegrini, María P. Morelli, Camila Martinena, Agustín Rolandelli, Florencia A. Castello, Nicolás Casco, Lorena M. Ciallella, Graciela C. de Casado, Rita Armitano, Juan Stupka, Claudio Gallego, Domingo J. Palmero, Verónica E. García, Nancy L. Tateosian

**Affiliations:** ^1^ Departamento de Química Biológica. Facultad de Ciencias Exactas y Naturales, UBA, Buenos Aires, Argentina; ^2^ Instituto de Química Biológica de la Facultad de Ciencias Exactas y Naturales (IQUIBICEN). Facultad de Ciencias Exactas y Naturales, Universidad de Buenos Aires. Consejo Nacional de Investigaciones Científicas y Técnicas, Buenos Aires, Argentina; ^3^ División Tisioneumonología, Hospital F.J. Muñiz, Buenos Aires, Argentina; ^4^ Hospital General de Agudos Parmenio Piñero, Buenos Aires, Argentina

**Keywords:** tuberculosis, active infection, intermediate and non-classical monocytes, myeloid-derived suppressor cells, anti-TB treatment

## Abstract

Alterations of myeloid cell populations have been reported in patients with tuberculosis (TB). In this work, we studied the relationship between myeloid-derived suppressor cells (MDSC) and monocytes subsets with the immunological responsiveness of TB patients. Individuals with active TB were classified as low responders (LR-TB) or high responders (HR-TB) according to their T cell responses against a cell lysate of *Mycobacterium tuberculosis* (*Mtb*-Ag). Thus, LR-TB, individuals with severe disease, display a weaker immune response to *Mtb* compare to HR-TB, subjects with strong immunity against the bacteria. We observed that LR-TB presented higher percentages of CD16 positive monocytes as compared to HR-TB and healthy donors. Moreover, monocyte-like (M-MDSC) and polymorphonuclear-like (PMN-MDSC) MDSC were increased in patients and the proportion of M-MDSC inversely correlated with IFN-γ levels released after *Mtb*-Ag stimulation in HR-TB. We also found that LR-TB displayed the highest percentages of circulating M-MDSC. These results demonstrate that CD16 positive monocytes and M-MDSC frequencies could be used as another immunological classification parameter. Interestingly, in LR-TB, frequencies of CD16 positive monocytes and M-MDSC were restored after only three weeks of anti-TB treatment. Together, our findings show a link between the immunological status of TB patients and the levels of different circulating myeloid cell populations.

## Introduction

Tuberculosis (TB) represents one of the main health global problems. The bacterial pathogen *Mycobacterium tuberculosis* (*Mtb*) was the second leading infectious killer after SARS-CoV-2 in 2020 (World Health Organization, 2021). Worldwide, an estimated 10.0 million people fell ill with TB and there were 1.5 million died because of this disease in 2020 (World Health Organization, 2021). Despite the use of the bacilli Calmette–Guérin (BCG) vaccine and the current chemotherapeutic treatments, TB remains as the top 13^th^ causes of death around the world (World Health Organization, 2021). Thus, one of the main challenges in developing new strategies to fight TB is focused on the reduction of the duration and complexity of drug regimens (World Health Organization, 2021). Therefore, there is a critical need to understand the mechanisms of immune control to achieve a significant impact on the epidemic (Scriba et al., 2008).

After infection, *Mtb* interacts with different cells of both, innate and adaptive immune compartments. These cells play an important role in the modulation and the development of the pathology (Bussi and Gutierrez, 2019). An efficient host protection against *Mtb* infection is associated with the induction, activation and proliferation of Th1 and Th17 cells (Jurado et al., 2012; du Plessis et al., 2013; Tateosian et al., 2017), whom promote the release of cytokines such as IL-2, TNF-α, IFN-γ, and IL-17; and the activation of effector monocytes (Jurado et al., 2012; du Plessis et al., 2013; Tateosian et al., 2017). Moreover, it has been demonstrated that reduced IFN-γ production is a marker of severe disease (Gong et al., 1996). Nevertheless, how *Mtb* is able to evade host immune surveillance and persist, particularly inside myeloid cells, is not fully elucidated yet. Inflammatory myeloid cells are key players in the pathophysiology of TB (Dorhoi and Kaufmann, 2015). In fact, it has been proposed that the phenotype of the populations of myeloid cells involved in early granuloma formation may influence substantially the progression of TB and the outcome of the infection (du Plessis et al., 2013; Guirado et al., 2013; Khan et al., 2019). Many studies have shown that *Mtb* can also affect the differentiation of progenitors and immature myeloid cells, the activation of recruited macrophages and shape the cytokine profile produced by them (Hickman et al., 2002; Khan et al., 2019). Furthermore, we have previously demonstrated that IFN-γ and IL-17A differentially regulate the autophagy process in *Mtb*-infected monocytes derived from TB patients in correlation with the severity of the disease (Rovetta et al., 2014; Tateosian et al., 2017). Phenotypically and functionally different subsets of monocytes were identified based on the relative expression of CD14 (co-receptor for toll-like receptor 4) and CD16 (Fc gamma receptor IIIa) (Ziegler-Heitbrock et al., 2010). Three subpopulations of human monocytes have been defined: classical (CD14^++^CD16^-^), intermediate (CD14^++^CD16^+^) and non-classical (CD14^+^CD16^++^), which may represent different stages of maturation (Ziegler-Heitbrock et al., 2010). During TB infection, CD16^+^ monocytes are expanded and do not differentiate into macrophages due to limited expression of maturation and differentiation markers such as CD11b, CD11c, CD33, and CD36 (Castaño et al., 2011). Besides, it has been described that the ability of circulating CD16^+^ monocytes to differentiate into dendritic cells and induce T-cell activation is decreased in TB patients (Balboa et al., 2011).

Myeloid-derived suppressor cells (MDSC) are another relevant population of phagocytes that participate in TB immunopathogenesis (Magcwebeba et al., 2019). Although little is known about the role of MDSC, accumulating evidence clearly demonstrates their capacity to suppress T-cell responses in TB (Magcwebeba et al., 2019). Moreover, MDSC have been reported to be linked to disease progression (du Plessis et al., 2013; Knaul et al., 2014; Tsiganov et al., 2014). MDSC described in TB mainly comprise two different subsets: monocyte-like MDSC (M-MDSC) and polymorphonuclear-like MDSC (PMN-MDSC) cells (Bronte et al., 2016; Kotzé et al., 2020). Although no specific markers have been described for MDSC identification (Magcwebeba et al., 2019), human M-MDSC were shown to express CD14, CD33, and CD11b with a lack of CD15 and low or no HLA-DR expression [CD11b^+^HLA-DR^-/low^(CD14^+^)] (Damuzzo et al., 2015; Bronte et al., 2016; Cassetta et al., 2019; Kotzé et al., 2020; Grassi et al., 2021). On the other hand, PMN-MDSC express CD15, CD33 and CD11b in the absence of CD14 and low or no HLA-DR expression [CD15^+^(CD14^-^CD11b^+^)] (Damuzzo et al., 2015; Bronte et al., 2016; Cassetta et al., 2019; Kotzé et al., 2020; Grassi et al., 2021).

The accumulation of MDSC during mycobacterial infections was first reported in mice models (Dietlin et al., 2007; Martino et al., 2010). Furthermore, the expansion of MDSCs was observed both in blood and in pleural fluid of patients with pulmonary or extra-pulmonary TB (du Plessis et al., 2013). Besides, it was also described a clear decrease of circulating MDSC frequencies at the end of successful anti-TB treatments (du Plessis et al., 2013).

Due to the important role of monocytes and MDSC during human *Mtb* infection, in this study, we hypothesized that the levels of these circulating myeloid cells in TB patients could be related to their immunological parameters and the influence of anti-TB therapy. Thus, we found a differential expansion profile of monocytes and MDSC according to the immunological status of TB patients. We could observe that low responder TB patients (LR-TB) presented higher percentages of circulating CD14^++^CD16^+^ and CD14^+^CD16^++^ monocytes and M-MDSC as compared to high responder patients (HR-TB) and healthy donors (HD). Furthermore, levels of CD14^++^CD16^+^ and CD14^+^CD16^++^ monocytes and M-MDSC were restored to normal after the first three weeks of anti-TB treatment in LR-TB. Therefore, we could show a relationship between the immunological status of TB patients and the frequencies of circulating CD16 positive monocytes and M-MDSC, suggesting that could be a new criterion to establish the immunological classification of TB patients. Taking into account the crucial role of cellular immunity during TB, these findings could be important pieces to better understand *Mtb* infection and TB disease.

## Materials and Methods

### Study Subjects

All individuals who participated in this study (HD and TB) had been vaccinated with BCG according to Argentine regulations. Patients with TB were diagnosed at Dr. F. Muñiz or Dr. P. Piñero Hospitals (Buenos Aires, Argentina) based on clinical and radiological data together with the identification of acid-fast bacilli (AFB) in sputum. First peripheral blood samples were collected between 1 and 7 days of anti-tuberculosis (anti-TB) therapy administration and second samples were obtained between 14 and 21 days of anti-TB treatment. All patients were treated with anti-TB regular therapy for drug sensitive *Mtb* strains, according to WHO and local guidelines [Asociacion Argentina de medicina respiratoria, 2009; World Health Organization and Stop TB Initiative (World Health Organization), 2010].

Bacillus Calmette-Guerin (BCG) vaccinated healthy donor individuals (HD) lacking a history of TB participated in this study.

Peripheral blood was collected in heparinized tubes from each participant after obtaining a written informed consent for the collection of samples and the subsequent analysis. All methods were carried out in accordance with relevant guidelines and regulations.

The protocols conducted in this work were approved by the Comité de Ética en Investigación, Hospital Parmenio Piñero, Ciudad de Buenos Aires, Argentina (Protocol Number: 594/17) and by Comité de Ética en Investigación, Hospital F.J. Muñiz, Ciudad de Buenos Aires, Argentina (Protocol Number: 1542/19).

### Exclusion Criteria and Classification of Patients

The exclusion criteria were carried out as previously described (Pellegrini et al., 2021). Briefly, individuals participating of the study were 18 - 60 years old and had no history of diseases affecting the immune system, such as HIV infection, treatment with immunosuppressive drugs, a recent diagnosis of cancer, hepatic or renal disease, pregnancy, or positive serology for other viral (e.g., hepatitis A, B or C), or bacterial (e.g., leprosy, syphilis) infections.

Individuals with latent infection were excluded from the present study by using the QuantiFERON-TB Gold Plus kit (Qiagen, Germany, USA).

TB Patients were classified as high responders (HR-TB) or low responders (LR-TB), based on their *in vitro* lymphocyte responses to a whole cell lysate of *M. tuberculosis* (*Mtb*-Ag) as previously described (Pasquinelli et al., 2004). Briefly, HR-TB patients are individuals displaying significant proliferative responses, IFN-γ production and an increased percentage of SLAMF1^+^ CD3^+^ cells after *Mtb*-Ag stimulation; whereas LR-TB patients exhibit low proliferative responses, IFN-γ release and SLAMF1^+^ CD3^+^ cells. LR-TB patients had more severe pulmonary disease compared with HR individuals. Cut-off values to differentiate between LR-TB and HR-TB were established previously by Pasquinelli et al (Pasquinelli et al., 2004). The fulfillment of two of these three criteria was sufficient to assign a patient to the corresponding group.

### Antigen

*In vitro* stimulation of fresh peripheral blood mononuclear cells (PBMC) was performed with a cell lysate from the virulent *Mycobacterium tuberculosis* strain H37Rv, prepared by probe sonication (*Mtb*-Ag) (BEI Resources, NIAID, NIH: *Mtb*, Strain H37Rv, Whole Cell Lysate, NR-14822).

### Cell Preparation and Reagents

All experiments were performed with fresh PBMC isolated by centrifugation on Ficoll-Hypaque (Amersham Biosciences, NJ, USA). Flow cytometry assays were performed after PBMC were washed with PBS plus 1% BSA and 0.1% NaN_3_ and resuspended in staining buffer [PBS plus 1% Fetal Bovine Serum (FBS)]. To evaluate cell viability, propidium iodide (PI) method was used in all experiments. PBMC were also cultured (1 × 10^6^ cells/mL), with or without *Mtb*-Ag (10 μg/mL) with RPMI 1640 medium (Gibco, MD, USA) supplemented with 1% L-glutamine, 1% penicillin/streptomycin, and 10% FBS (Gibco, MD, USA) during 48 h. Monocyte absolute number was evaluated by routine blood count.

### Flow Cytometry

Fresh PBMC from TB patients and HD were stained with specific fluorophore-marked antibodies against CD14 (FITC, clone HCD14, BioLegend, USA) and CD16 (APC, clone 3G8, BioLegend, USA) for differentiating monocyte subsets. Evaluation of MDSC percentage was performed using PBMC stained with specific fluorophore-marked antibodies against CD14 (FITC, clone HCD14, BioLegend, USA), CD11b (PE, clone ICRF44, BioLegend, USA), CD15 (PE/Cy7, clone HI98, BioLegend, USA), and HLA-DR (APC, clone L243, BioLegend, USA). Negative control samples were incubated with irrelevant isotype matched monoclonal antibody (FITC Mosue IgG1κ, APC Mouse IgG1κ, PE/Cy7 Mouse IgMκ and APC Mouse IgG2aκ isotype ctrl antibodies, BioLegend, USA) (**Supplementary Figure 1**). Cell viability was evaluated by PI method. All samples were analyzed on a FACSAria II flow cytometer (BD Biosciences, CA, USA).

### IFN-γ Determination

The levels of IFN-γ were evaluated in supernatants using a commercial ELISA kit (Human IFN-γ ELISA MAX Standard Kit, BioLegend, USA) following the manufacturer´s instructions.

IFN-γ index: Fresh PBMC were stimulated with *Mtb*-Ag for 48h and then supernatants were obtained. IFN-γ was measured by ELISA and the index for each individual was calculated as (pg/mL_IFN-γ_ after *Mtb*-Ag stimulation)/(pg/mL_IFN-γ_ after culturing with medium).

### Proliferation Index

Fresh PBMC were stimulated with *Mtb*-Ag for five days and then, cells were pulsed with [^3^H]TdR (1 μCi/well) and harvested 16 hours later. [^3^H]TdR incorporation was measured in a liquid scintillation counter as counts per minute (c.p.m). Proliferation index for each individual was calculated as (c.p.m. after *Mtb*-Ag stimulation)/(c.p.m. after culturing with medium).

### Immunosuppression Functional Assay

M-MDSC from TB patients were obtained from fresh PBMC by cell sorting (FACS). Cells were labeled with anti-CD14 (FITC, clone HCD14, BioLegend, USA), anti-CD11b (PE, clone ICRF44, BioLegend, USA) and anti-HLA-DR (APC, clone L243, BioLegend, USA) and then sorted *via* a FACSAria II flow cytometer (BD Biosciences, CA, USA). FACS-sorted M-MDSC were co-cultured with autologous lymphocytes (ratio 1:1) and treated with anti-CD3 (0.1µg/mL, clone OKT3, Biolegend, USA) and anti-CD28 (0.5µg/mL, clone CD28.8, Biolegend, USA) antibodies. Stimulated lymphocytes without adding M-MDSC were used as control. Each condition was run in triplicate. Then supernatants were collected after 48h and IFN-γ was measured by ELISA. Relative IFN-γ release was calculated to Control (100%). Furthermore, proliferation was measured by pulsing cultured cells with [^3^H]TdR (1 μCi/well) at day 5 and harvested 16 hours later. [^3^H]TdR incorporation was measured in a liquid scintillation counter as counts per minute (c.p.m). Relative proliferation was calculated to Control (100%).

### Statistical Analysis

Analysis of variance (ANOVA) and *post hoc* Tukey´s multiple comparisons test were used as indicated in figure legends. The Mann–Whitney *U* test and the Wilcoxon matched-pairs signed-rank were used to analyze differences between groups. For categorical variables, the Chi-square (and Fisher´s exact) test for homogeneity was performed to compare proportions of subjects between groups. In the indicated graphs each symbol represents an individual and the horizontal lines indicate the mean ± standard deviation (SD). Correlations were calculated using the non-parametric Spearman correlation test. Receiver operating characteristic (ROC) curve analysis was performed to analyze the predictive value of the frequencies of CD14^+^ **^+^** CD16^+^, CD14^+^CD16^++^ and M-MDSC cells populations, calculating the area under the curve (AUC) and the 95% confidence interval (CI). Analyses were performed using GraphPad Prism 8.0.2 software. *P* < 0.05 was considered statistically significant.

## Results

**Figure 1:**
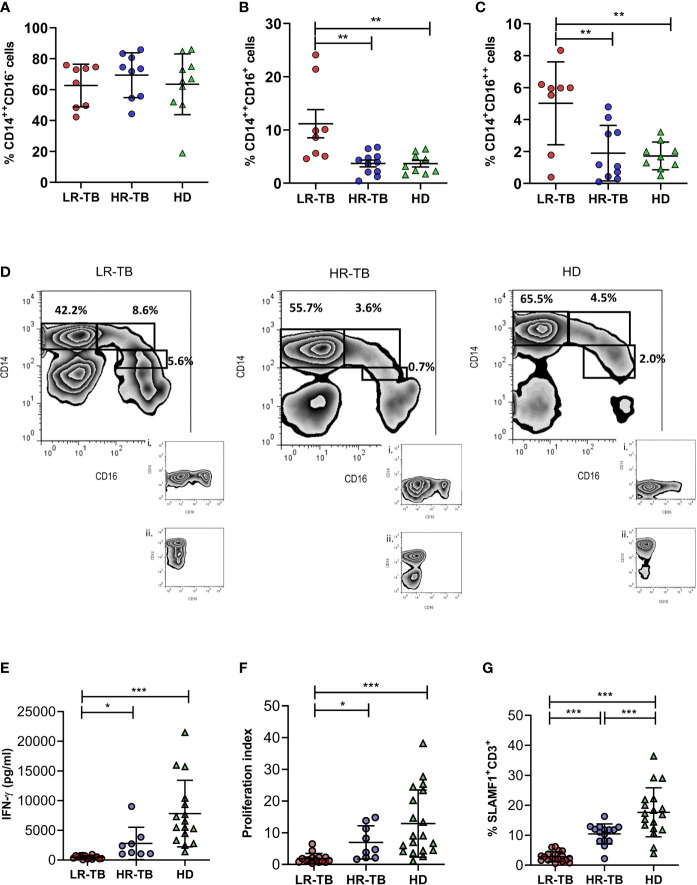
Monocyte profile and immunological classification parameters of TB patients. Monocyte subpopulation frequencies in peripheral blood mononuclear cell fraction from low responder TB patients (LR-TB), high responder TB patients (HR-TB) and healthy donors (HD) were determined by flow cytometry. Percentages of CD14^++^CD16^-^ **(A)**, CD14^++^CD16^+^ **(B)** and CD14^+^CD16^++^ **(C)** monocytes are shown. Mean of percentages of cells ± SD for each group of subjects are shown (LR-TB N = 8, HR-TB N = 11 and HD N = 10). *P* values were calculated by one-way ANOVA and Tukey´s multiple comparison post-test for unpaired samples. **P < 0.01. Each symbol represents an individual. A representative density plot and isotype controls i) for CD14 FICT and ii) for CD16 APC of each group of individuals is shown **(D)**. **(E)** IFN-γ levels measured by ELISA in supernatants of fresh PBMC stimulated for 48h with *Mtb*-Ag (10 µg/ml). **(F)** Proliferation index evaluated by [^3^H]-thymidine incorporation. (Proliferation index = c.p.m. after 5 days of *Mtb*-Ag-stimulation/c.p.m. after unstimulation). **(G)** Increase in % of SLAMF1^+^ T cells. Fresh PBMC were stimulated with *Mtb*-Ag (10 µg/ml) for 5 days. Afterwards, the expression of SLAMF1^+^ on CD3^+^ lymphocytes were determined by flow cytometry. Mean of the percentage of cells ± SD for each group is shown. *P < 0.05, **P < 0.01, ***P < 0.001. Each symbol represents an individual. For **(E–G)** LR-TB N = 19, HR-TB N = 15 and HD N = 19. *P* values were calculated by one-way ANOVA and Tukey´s multiple comparison post-test for unpaired samples.

We initially investigated the subsets of circulating blood monocytes in pulmonary TB patients classified according to their immunological responsiveness against *Mtb*-Ag. As previously described in other populations, TB patients showed higher percentage of circulating CD16^+^ monocytes than healthy BCG-vaccinated donors (HD) (TB = 10.1% ± 1.9; HD = 5.4% ± 0.8; Mean ± SD. *P < 0.05, Mann–Whitney *U* test). Furthermore, percentages of classical (CD14^++^CD16^-^), intermediate (CD14^++^CD16^+^) and non-classical (CD14^+^CD16^++^) monocytes were also analyzed in two groups of TB patients classified as high responders (HR-TB) or low responders (LR-TB) based on their *in vitro* lymphocyte responses against *Mtb*-Ag as previously described (Pasquinelli et al., 2004). Demographic characteristics, clinical and immunological parameters of the studied populations are shown in (**Table 1**). Our results showed no perturbation of classical monocytes percentages when comparing LR-TB, HR-TB and HD (**Figure 1A**). On the other hand, we found that LR-TB patients presented the highest percentages of CD14^++^CD16^+^ (**Figure 1B**) and CD14^+^CD16^++^ (**Figure 1C**) circulating cells as compared to HR-TB and HD subjects. Moreover, HR-TB patients showed similar frequencies of CD14^++^CD16^+^ and CD14^+^CD16^++^ to HD. Representative flow cytometry plots are shown in **Figure 1D**. Nevertheless, it is important to mention that the analysis of total monocyte count in peripheral blood showed no differences between LR-TB and HR-TB (**Supplementary Figure 2**). The HR-TB and LR-TB sub-populations of patients were characterized on their *in vitro* lymphocyte responses against *Mtb*-Ag as previously described in the Methods section. **Figures 1E–G** show differential levels of IFN-γ, proliferation index and percentages of SLAMF1^+^CD3^+^ cells in the two groups of TB patients and HD after stimulating their PBMC with the *Mtb*-Ag. In view of our results, and to evaluate the potential use of CD14^++^CD16^+^ and CD14^+^CD16^++^ cells frequencies as new criteria to discriminate individuals LR-TB from HR-TB, we performed ROC analyses. From this study, significant results were obtained for CD14^++^CD16^+^ cells (AUC =0.9091; *P* < 0.01; 95% CI: 0.7788–1.000) and for CD14^+^CD16^++^ cells (AUC =0.8500; *P* < 0.01; 95% CI: 0.6437–1.000), demonstrating that the percentages of circulating intermediate and non-classical monocytes allow differentiating between these patients with distinct immunological status. A cut-off value of 5.0% for CD14^++^CD16^+^ cells allowed a differentiation with a Sensitivity =87.5% and a Specificity =81.8%. Moreover, for CD14^+^CD16^++^ a cut-off value of 5.2% allowed a differentiation with a Sensitivity =75.0% and a Specificity =100%. Therefore, the analysis of the different monocyte subsets could serve as new criteria for the immunological classification of TB patients.

**Table 1:** Demographic characteristics, clinical and immunological parameters of tuberculosis patient and healthy donor populations.

		LR-TB	HR-TB	HD	*P*-value
**N**		23	20	19	-------
**Age**		26.7 ± 1.3	25.3 ± 2.5	29.6 ± 1.1	0.103^a^
**Sex**	**Male**	55.6	53.4	51.6	
	**Female**	44.4	46.6	48.4	0.941^b^
**IFN-γ index**		29.7 ± 6.0	154.6 ± 53.7	217.9 ± 46.0	<0.0001^a^
**Proliferation index**		1.9 ± 0.4	6.9 ± 1.7	12.9 ± 2.4	0.0006^a^
**Increase in the % of SLAMF1^+^ T cells**		3.8 ± 4.8	10.3 ± 3.4	17.6 ± 1.9	<0.0001^a^
**Lymphocytes (%)**		14.6 ± 4.7	16.9 ± 1.6	28.4 ± 1.0	0.003^a^
**Monocytes (%)**		8.3 ± 1.1	9.8 ± 1.3	6.4 ± 0.5	0.227^a^
**Neutrophils (%)**		61.9 ± 7.5	62.2 ± 6.3	61.5 ± 1.2	0.941^a^
**Time of Disease Evolution (days)**		85 ± 12.9	41.2 ± 10.3	------	0.013^c^
**AFB in sputum smear (%)**	(+)	94.8	93.3	------	0.999^b^
	(-)	5.2	6.7	------	

TB patients were classified as LR-TB and HR-TB according to IFN-γ index; proliferation index and increase in the percentage of SLAMF1^+^-positive T cells in response to Mtb-Ag stimulation. The fulfillment of two of these three criteria was sufficient to assign a patient to the corresponding group. Proportion of lymphocytes, monocytes and neutrophils are shown as percentages of total white blood cells. Time of disease evolution (days) was established by analyzing the following clinical symptoms in patients previous to hospital admission: weight loss, night sweats, symptoms of malaise or weakness, persistent fever, presence of cough, history of shortness of breath, and/or hemoptysis. Mean ± SEM are shown for continuous data. Categorical data (Sex and AFB in sputum smear) are expressed as percentages. ^a^P values were calculated by ANOVA test. ^b^P values were calculated by Chi-Square (and Fisher´s exact) test for categorical variables. ^c^P value was calculated by Mann–Whitney U test. P values < 0.05 were considered significant. HD, Healthy donors (BCG vaccinated); LR-TB, Low responder tuberculosis patients; HR-TB, High responder tuberculosis patients; AFB, Acid-Fast Bacilli.

**Figure 2:**
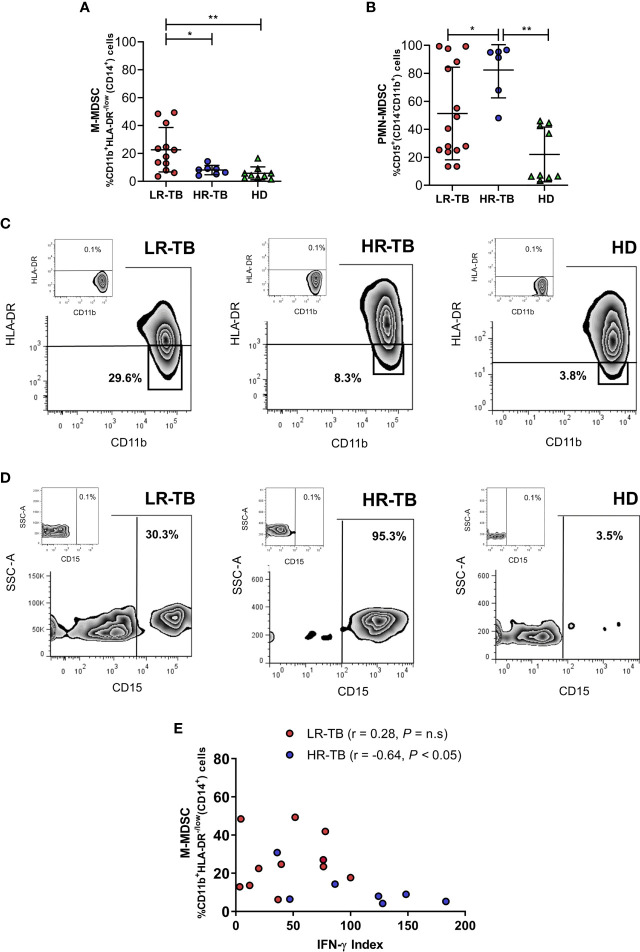
Myeloid derived suppressor cells in patients with active tuberculosis. The frequencies of **(A)** M-MDSC [%CD11b^+^HLA-DR^-/low^(CD14^+^)] cells and **(B)** PMN-MDSC [%CD15^+^ (CD14^-^CD11b^+^)] cells were determined by flow cytometry in PBMC from LR-TB, HR-TB and HD. Mean of the percentage of cells ± SD for each group is shown. *P < 0.05, **P < 0.01. For **(A)** LR-TB N = 12, HR-TB N = 7 and HD = 9 and **(B)** LR-TB N = 15, HR-TB N = 6 and HD N = 9. Each symbol represents an individual. A representative density plot for M-MDSC **(C)** and PMN-MDSC **(D)** of each group of individuals is shown. **(E)** Correlations between the IFN-γ index and percentages of circulating M-MSDC in LR-TB and HR-TB patients. Fresh PBMC from TB patients were stimulated with *Mtb*-Ag (10 µg/ml) for 48 h Afterwards, the production of IFN-γ was determined in supernatants by ELISA and the IFN-γ index was calculated as described. Correlation factor (r) and *P* values were calculated using the Spearman correlation test (LR-TB N = 12 and HR-TB N = 7). *P < 0.05.

Then, we also decided to compare the frequencies of circulating MDSC in our study population. We observed significantly higher levels of monocyte-like MDSC (M-MDSC) and polymorphonuclear-like MDSC (PMN-MDSC) in TB patients when compared with HD (**Supplementary Figures 3A, B**). To further investigate the clinical significance of MDSC in TB disease, we studied whether LR-TB, HR-TB and HD presented different levels of circulating M-MDSC and PMN-MDSC. The FACS analysis showed higher levels of M-MDSC in LR-TB in comparison with HR-TB and HD individuals (**Figure 2A**). On the contrary, HR-TB patients presented the highest levels of PMN-MDSC compared to LR-TB and HD (**Figure 2B**). Representative flow cytometry plots are shown in **Figure 2C** (M-MDSC) and **Figure 2D** (PMN-MDSC). Then, due to the importance of IFN-γ in the immune response against *Mtb*, we evaluated the possibility of a correlation between percentages of MDSC and the IFN-γ index in the two TB patient populations. The performed analysis did not show a correlation in the percentages of PMN-MDSC for any of the TB patient sub-populations. Furthermore, no correlation for LR-TB was observed between M-MDSC and IFN-γ index; however, it was observed that the percentage of M-MDSC negatively correlated with IFN-γ index in HR-TB patients. (**Figure 2E**). These results suggested an immunosuppressive role of M-MDSC on TB patients´ lymphocytes. To corroborate this, functional experiments were performed. We investigated the suppressive potential of M-MDSC from recently diagnosed active TB patients on T-cell function by analyzing proliferation and IFN-γ production. Co-culture experiments showed that sorted CD14^+^CD11b^+^HLA-DR^-/low^ from TB patients exhibit the ability to suppress polyclonally stimulated (anti-CD3, anti-CD28) autologous lymphocytes proliferation and the production of IFN-γ (**Supplementary Figures 4A, B**). Thus, we could confirm the suppressive potential of these M-MDSC from TB patients.

Moreover, in order to assess the potential use of circulating M-MDSC frequencies as a new criterion for discriminating between LR-TB and HR-TB, we next performed a ROC analysis, which showed significant results (AUC =0.7857; *p* < 0.05; 95% CI: 0.58–0.99; Cut-off =11.0%; Sensitivity =75.0%; Specificity =85.7%). This demonstrated that, together with the percentages values observed for circulating intermediate and non-classical monocytes, M-MDSC frequencies could be used also as another immunological classification parameter of disease severity.

**Figure 3:**
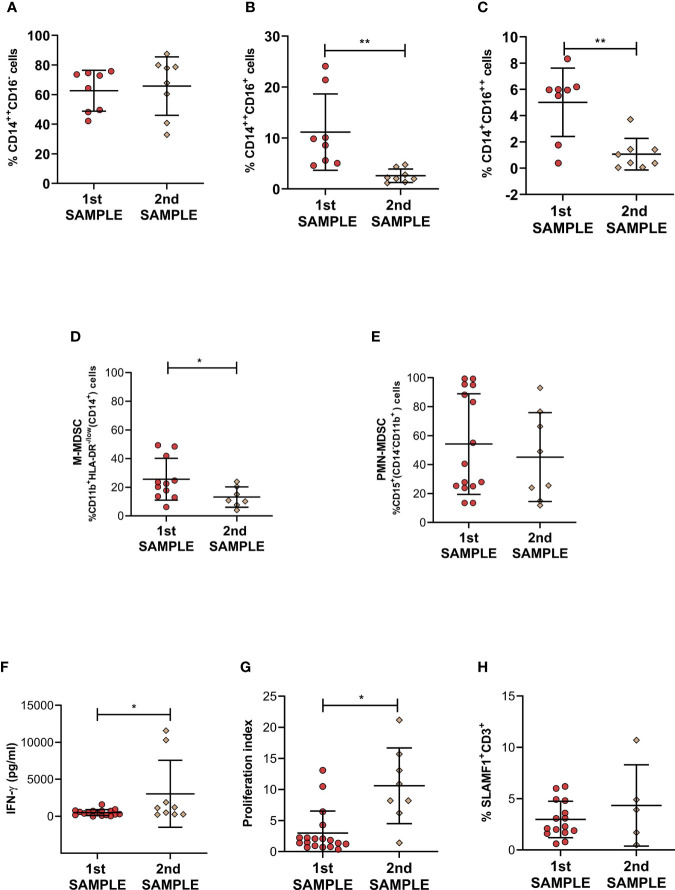
Monocytes and Myeloid derived suppressor cells in active LR-TB patients undergoing anti-TB treatment. The frequencies of **(A)** CD14^++^CD16^-^, **(B)** CD14^++^CD16^+^, **(C)** CD14^+^CD16^++^ monocytes, **(D)** M-MDSC [%CD11b^+^HLA-DR^-/low^(CD14^+^)] and **(E)** PMN-MDSC [%CD15^+^(CD14^-^CD11b^+^)] were determined by flow cytometry in fresh PBMC from LR- TB patients at the beginning of regular anti-TB treatment (blood samples taken during the first week of chemotherapy - first sample) and after 14 - 21 days (second sample). **(F)** IFN-γ levels measured by ELISA in supernatants of PBMC obtained as first or second sample and stimulated for 48h with *Mtb*-Ag (10 µg/ml). **(G)** Proliferation index evaluated by [^3^H]-thymidine incorporation. **(H)** SLAMF1 on CD3^+^ lymphocytes were determined by flow cytometry in fresh PBMC obtained as first or second sample. For **(A–C)**: 1^st^ sample N=8 and 2^nd^ sample N=8, for **(D)**: 1^st^ sample N=12 and 2^nd^ sample N=7, for **(E)**: 1^st^ sample N=15 and 2^nd^ sample N=8, for **(F)**: 1^st^ sample N=18 and 2^nd^ sample N=9, for **(G)**: 1^st^ sample N=15 and 2^nd^ sample N=5 and for **(H)**: 1^st^ sample N=15 and 2^nd^ sample N=5. *P* values were calculated by Mann-Whitney *U* test for unpaired samples. Each symbol represents an individual. Mean values ± SD are shown. *P < 0.05, **P < 0.01.

It was previously described a significant reduction in MDSC frequencies in patients with TB at the end of antibiotic treatment; therefore, we decided to study whether that effect could be observed after a few weeks of therapy in TB patients with a diminished immune responsiveness against *Mtb*. We then compared percentages of blood circulating PMN-MDSC, M-MDSC and classical, intermediate and non-classical monocytes at the beginning and after three weeks of anti-TB treatment. No differences in the percentage of CD14^++^CD16^-^ were observed after this treatment period in LR-TB patients. However, we observed a significant decrease in CD14^++^CD16^+^ and CD14^+^CD16^++^ monocyte levels after this short period of treatment (**Figures 3A–C**). In addition, in LR-TB patients, we observed a significant reduction of the frequency of M-MDSC (**Figure 3D**). In contrast, in the same group of patients the percentage of PMN-MDSC was not affected by the anti-TB treatment (**Figure 3E**). Representative flow cytometry plots are shown in **Supplementary Figure 5**. At the same time that these modifications were detected in LR-TB cell populations an increase in IFN-γ production and proliferation index was also observed after PBMCs stimulation with *Mtb*-Ag (**Figures 3F, G**). However, no differences were detected in the percentage of SLAMF1^+^CD3^+^ T cells (**Figure 3H**). It is important to point out that none of these parameters were modified during this short period of time in HR-TB patients.

Together, our present findings extend the knowledge about TB patient immunological parameters and how it is affected by a few days of anti-TB treatment. The accumulation of intermediate and non-classical monocytes and M-MDSC in LR-TB patients suggests a differential role of this cells populations in TB patients with a weaker immune response.

## Discussion

A spectrum of stages caused by *Mtb* infection leads to identify that some patients can control the bacterial infection and others cannot. The IFN-γ producing Th1 cells are essential to control mycobacterial replication (Serbina et al., 2001; Salgame, 2005). Indeed, reduced IFN-γ production is a well-known marker of disease severity (Gong et al., 1996). In our study population, HR-TB and LR-TB patients were identified based on their T cell responses against *Mtb-*Ag *(* Pasquinelli et al., 2004). Th1 and Th17 cells alone do not explain the resistance/susceptibility to infection and disease (Forbes et al., 2008; Jurado et al., 2012), suggesting that other actors might be required during the immune regulation of TB. Myeloid cells are a heterogeneous group of cells that plays a major role in the regulation of immune responses in many pathological conditions. However, the imbalance of myeloid cells during human TB has poorly been studied. Therefore, we aimed to investigate populations of monocytes and MDSC cells during human active TB disease. In the present report, we identified a specific profile in different circulating myeloid cell populations in TB patients. Our novelty data show that TB patients with weakened immune responses linked to high levels of peripheral blood intermediate and non-classical monocytes and M-MDSC. However, no differences were observed in classical monocytes and PMN-MDSC. Moreover, we present for the first time to our knowledge data showing the effect of short-term anti-TB treatment on these cell populations in LR-TB patients. We found that patients showing weak or no levels of lymphocytes proliferation, IFN-γ production and SLAMF1 positive T cells after stimulation with *Mtb*-Ag, presented higher percentages of intermediate and non-classical monocytes as compared to HR-TB and HD (**Figure 1**). Moreover, LR-TB individuals also presented the highest percentages of circulating M-MDSC. On the contrary, HR-TB individuals displayed the highest percentages of PMN-MDSC (**Figure 2**). These results are clearly in agreement with those recently described by Grassi et al. where an association between PMN-MDSC levels and the severity of TB disease, evaluated by chest X-ray, was demonstrated (Grassi et al., 2021). In that work, the authors show that the frequencies of PMN-MDSC are higher in those TB patients classified with a low/mild severity score compared to those classified with a high severity score. However, it is important to mention that this classification criterion does not take into account immunological parameters (Grassi et al., 2021). We also observed for the first time, that the frequencies of CD14^++^CD16^+^ and CD14^+^CD16^++^ monocytes and M-MDSC return close to healthy control levels after a short time of anti-TB treatment (**Figure 3**). Therefore, we showed that these cell subsets could contribute to a better characterization of the immunological profile of TB patients and could be new targets for the development of host-directed therapies.

Previously, we have described that monocytes from LR-TB individuals were unable to activate the autophagy process through IL-17A at least in part because of a defect in the MAPK1/3 signaling pathway. In contrast, both IFN-γ and IL-17A increased the levels of autophagy in patients with strong immunity to *Mtb* (Tateosian et al., 2017). Moreover, circulating CD16^+^ monocytes and M-MDSC have been associated with clinical parameters of TB disease severity (Sampath et al., 2018; Jøntvedt Jørgensen et al., 2020). Sampath et al., have found that CD16 positive monocytes are expanded in TB infection and perturbation of this subset defines the severity of TB (Sampath et al., 2018). More recently, Jorgensen et al. have shown that M-MDSC frequencies correlated with TB disease severity by evaluating cavitary disease, erythrocyte sedimentation rate and monocyte:lymphocyte ratio among other parameters (Jøntvedt Jørgensen et al., 2020). Furthermore, it has been previously described a positive correlation between PMN-MDSC frequencies and percentage of monocytes in *Mtb* infected subjects (TB and LTBI). However, this correlation was observed in CD14^++^CD16^-^ but not in CD14^-^CD16^++^ and CD14^++^CD16^+^ monocytes (Grassi et al., 2021). Our present results extended those findings, demonstrating that LR-TB patients, individuals with weakened immune response (Pasquinelli et al., 2004; Jurado et al., 2012), showed higher proportions of intermediate and non-classical monocytes and M-MDSC in peripheral blood as compared to HR-TB patients, individuals with a strong immunity in response to *Mtb*-Ag. Thus, the proportion of circulating CD14^++^CD16^+^ and CD14^+^CD16^++^ monocytes and M-MDSC in patients with active TB might be also reflecting the immunological and clinical severity of the disease.

In accordance with previous reports (du Plessis et al., 2013; Zhan et al., 2018), we found that MDSC are expanded in peripheral blood of active TB patients (**Supplementary Figure 3**). Moreover, we show for the first time that there is a characteristic expansion profile of M-MDSC and PMN-MDSC in TB patients related to their immunological status and responsiveness. Our results in fresh PBMC confirmed that the majority of HR-TB patients present augmented percentages of PMN-MDSC. Furthermore, as we mentioned before, patients with weak immunity to *Mtb* presented the highest levels of circulating M-MDSC. However, the inverse correlation between the percentages of circulating M-MDSC and IFN-γ index was only observed in HR-TB patients (**Figure 2**). These would suggest a marked suppressive effect of M-MDSC from HR-TB than cells from LR-TB. Moreover, these results are in agreement with those reported previously where a functional correlation between MDSC and TB infection was also suggested (Lee et al., 2012; du Plessis et al., 2013). Several studies have recently reported the function and the accumulation of MDSC in association with pathogen load (du Plessis et al., 2013; Knaul et al., 2014). du Plessis, et al. showed an increased frequency of MDSC during active TB with suppressed T cell functions (du Plessis et al., 2013). Furthermore, in a mice model, Knaul, et al. described that MDSC are able to phagocyte *Mtb*, and released both, pro-inflammatory (IL-6, IL-1α) and immunomodulatory (IL-10) cytokines while retaining their suppressive capacity (Knaul et al., 2014). Moreover, it was reported that transmembrane-TNF expressed on MDSC is crucial for its suppressive activity that regulates the inflammatory process associated with *Mtb* infection (Chavez-Galan et al., 2017). Both, HR-TB and LR-TB patients showed a similar number of AFB in sputum smear, indicating no differences in the bacterial load between them. This could suggest that the immune microenvironment generated during the chronic inflammation of *Mtb* infection might influence the accumulation and function of monocytes and MDSC.

Additionally, it has been previously shown that pharmacological therapy reduce the accumulation of the myeloid cell population (Sánchez et al., 2006; du Plessis et al., 2013; Zhan et al., 2018). Sanchez et al. demonstrated that the expression of CD14, HLA-DR and CD36 was decreased in monocytes of TB patients (Sánchez et al., 2006); nevertheless, normal expression of these molecules was restored after 6 months of anti-TB treatment. Furthermore, in TB patients, the MDSC population was reduced at the end of anti-TB therapy (du Plessis et al., 2013; Zhan et al., 2018). However, it is important to mention that in our study we could observe for the first time to our knowledge, a restoration in the circulating levels of CD14^++^CD16^+^ and CD14^+^CD16^++^ monocytes and M-MDSC after a short term (three weeks) of anti-TB treatment in LR-TB. In this short period, we did not observe differences in percentages of circulating CD14^++^CD16^-^ or in PMN-MDSC levels. Further investigation in studies with larger patient numbers was required. Therefore, considering previously published data and our present results, we hypothesize that intermediate and non-classical monocytes and M-MDSC populations could serve as a treatment-response marker during TB. In addition, the circulating levels of CD14^++^CD16^+^ and CD14^+^CD16^++^ monocytes and M-MDSC, together with information about the immunological status of patients related to T cell responses, would contribute to differentiate more efficiently between LR-TB and HR-TB patients.

## Data Availability Statement

The raw data supporting the conclusions of this article will be made available by the authors, without undue reservation.

## Ethics Statement

The studies involving human participants were reviewed and approved by Hospital F.J. Muñiz and Hospital P. Piñero ethics committes. “Estudio de los mecanismos inmunológicos que operan durante la infección humana por *Mycobacterium tuberculosis* conduciendo a enfermedad activa o contención del patógeno” (approved by the Ethics Committee of Hospital F.J. Muñiz) and “Desarrollo de un nuevo método de detección de infección con *Mycobacterium tuberculosis* basado en tetrámeros CMH- peptidos de antígenos de latencia” (approved by the Ethics Committe of Hospital P. Piñero). The patients/participants provided their written informed consent to participate in this study.

## Author Contributions

NT designed the study. NA, NT, JMP, MM, CM, AR, and FC were responsible for performing flow cytometry analysis, ELISA, and proliferation assays. NA was in charge of performing the QFT test in HD to evaluate latent infection. NA, NT, JMP, MM, and VG did the data management and analysis. NT, NA, JMP and MM prepared all the figures and tables. JMP, MM, AR, CM, and FC were responsible for processing samples and contributed with standard laboratory work. NA and NT wrote the manuscript. VG provided also expert advice. NC, LC, GC, RA, JS, CG, and DP were in charge of patient recruitment, diagnosis of active tuberculosis, and sample collection. All authors contributed to data gathering and interpretation, and revision of the report.

## Funding

This work was supported by grants from Agencia Nacional de Promoción Científica y Tecnológica (ANPCyT) (PICT2017-1158 and PICT2014-1709 to NT, PICT 2017-1451 to VG and PICT Start Up 2016-0022 to NA) and Universidad de Buenos Aires (UBACyT) (20020170100127BA to VG and 200201150200107BA to NA).

## Conflict of Interest

The authors declare that the research was conducted in the absence of any commercial or financial relationships that could be construed as a potential conflict of interest.

## Publisher’s Note

All claims expressed in this article are solely those of the authors and do not necessarily represent those of their affiliated organizations, or those of the publisher, the editors and the reviewers. Any product that may be evaluated in this article, or claim that may be made by its manufacturer, is not guaranteed or endorsed by the publisher.

## References

[B1] Asociacion Argentina de medicina respiratoria (2009). Consenso Argentino De Tubeculosis (Buenos Aires: AAMR). Available at: https://www.aamr.org.ar/recursos_educativos/consensos/consenso_tbc_aamr_29_01_09.pdf.

[B2] Balboa L. Romero M. M. Basile J. I. Sabio y García C. A. Schierloh P. Yokobori N.. (2011). Paradoxical Role of CD16+CCR2+CCR5+ Monocytes in Tuberculosis: Efficient APC in Pleural Effusion But Also Mark Disease Severity in Blood. J. Leukoc. Biol. 90 (1), 69–75. doi: 10.1189/jlb.1010577 21454357

[B3] Bronte V. Brandau S. Chen S.-H. Colombo M. P. Frey A. B. Greten T. F.. (2016). Recommendations for Myeloid-Derived Suppressor Cell Nomenclature and Characterization Standards. Nat. Commun. 7, 12150. doi: 10.1038/ncomms12150 27381735 PMC4935811

[B4] Bussi C. Gutierrez M. G. (2019). Mycobacterium Tuberculosis Infection of Host Cells in Space and Time. FEMS Microbiol. Rev. 43 (4), 341–361. doi: 10.1093/femsre/fuz006 30916769 PMC6606852

[B5] Cassetta L. Baekkevold E. S. Brandau S. Bujko A. Cassatella M. A. Dorhoi A.. (2019). Deciphering Myeloid-Derived Suppressor Cells: Isolation and Markers in Humans, Mice and Non-Human Primates. Cancer Immunol. Immunother. CII 68 (4), 687–697. doi: 10.1007/s00262-019-02302-2 30684003 PMC6447515

[B6] Castaño D. García L. F. Rojas M. (2011). Increased Frequency and Cell Death of CD16+ Monocytes With Mycobacterium Tuberculosis Infection. Tuberc Edinb Scotl 91 (5), 348–360. doi: 10.1016/j.tube.2011.04.002 21621464

[B7] Chavez-Galan L. Vesin D. Uysal H. Blaser G. Benkhoucha M. Ryffel B.. (2017). Transmembrane Tumor Necrosis Factor Controls Myeloid-Derived Suppressor Cell Activity *via* TNF Receptor 2 and Protects From Excessive Inflammation During BCG-Induced Pleurisy. Front. Immunol. 8, 999. doi: 10.3389/fimmu.2017.00999 28890718 PMC5574880

[B8] Damuzzo V. Pinton L. Desantis G. Solito S. Marigo I. Bronte V.. (2015). Complexity and Challenges in Defining Myeloid-Derived Suppressor Cells. Cytometry B Clin. Cytom 88 (2), 77–91. doi: 10.1002/cytob.21206 25504825 PMC4405078

[B9] Dietlin T. A. Hofman F. M. Lund B. T. Gilmore W. Stohlman S. A. van der Veen R. C. (2007). Mycobacteria-Induced Gr-1+ Subsets From Distinct Myeloid Lineages Have Opposite Effects on T Cell Expansion. J. Leukoc. Biol. 81 (5), 1205–1212. doi: 10.1189/jlb.1006640 17307863

[B10] Dorhoi A. Kaufmann S. H. E. (2015). Versatile Myeloid Cell Subsets Contribute to Tuberculosis-Associated Inflammation. Eur. J. Immunol. 45 (8), 2191–2202. doi: 10.1002/eji.201545493 26140356

[B11] du Plessis N. Loebenberg L. Kriel M. von Groote-Bidlingmaier F. Ribechini E. Loxton A. G.. (2013). Increased Frequency of Myeloid-Derived Suppressor Cells During Active Tuberculosis and After Recent Mycobacterium Tuberculosis Infection Suppresses T-Cell Function. Am. J. Respir. Crit. Care Med. 188 (6), 724–732. doi: 10.1164/rccm.201302-0249OC 23885784

[B12] Forbes E. K. Sander C. Ronan E. O. McShane H. Hill A. V. S. Beverley P. C. L.. (2008). Multifunctional, High-Level Cytokine-Producing Th1 Cells in the Lung, But Not Spleen, Correlate With Protection Against Mycobacterium Tuberculosis Aerosol Challenge in Mice. J. Immunol. Baltim Md 1950 181 (7), 4955–4964. doi: 10.4049/jimmunol.181.7.4955 PMC2867031 18802099

[B13] Gong J. H. Zhang M. Modlin R. L. Linsley P. S. Iyer D. Lin Y.. (1996). Interleukin-10 Downregulates Mycobacterium Tuberculosis-Induced Th1 Responses and CTLA-4 Expression. Infect. Immun. 64 (3), 913–918. doi: 10.1128/iai.64.3.913-918.1996 8641800 PMC173856

[B14] Grassi G. Vanini V. De Santis F. Romagnoli A. Aiello A. Casetti R.. (2021). PMN-MDSC Frequency Discriminates Active Versus Latent Tuberculosis and Could Play a Role in Counteracting the Immune-Mediated Lung Damage in Active Disease. Front. Immunol. 12, 594376. doi: 10.3389/fimmu.2021.594376 33981297 PMC8107479

[B15] Guirado E. Schlesinger L. S. Kaplan G. (2013). Macrophages in Tuberculosis: Friend or Foe. Semin. Immunopathol. 35 (5), 563–583. doi: 10.1007/s00281-013-0388-2 23864058 PMC3763202

[B16] Hickman S. P. Chan J. Salgame P. (2002). Mycobacterium Tuberculosis Induces Differential Cytokine Production From Dendritic Cells and Macrophages With Divergent Effects on Naive T Cell Polarization. J. Immunol. 168 (9), 4636–4642. doi: 10.4049/jimmunol.168.9.4636 11971012

[B17] Jøntvedt Jørgensen M. Jenum S. Tonby K. Mortensen R. Walzl G. Du Plessis N.. (2020). Monocytic Myeloid-Derived Suppressor Cells Reflect Tuberculosis Severity and Are Influenced by Cyclooxygenase-2 Inhibitors. J. Leukoc. Biol 110 (1), 177–186. doi: 10.1002/JLB.4A0720-409RR 33155730 PMC8359170

[B18] Jurado J. O. Pasquinelli V. Alvarez I. B. Peña D. Rovetta A. I. Tateosian N. L.. (2012). IL-17 and IFN-γ Expression in Lymphocytes From Patients With Active Tuberculosis Correlates With the Severity of the Disease. J. Leukoc. Biol. 91 (6), 991–1002. doi: 10.1189/jlb.1211619 22416258 PMC3360475

[B19] Khan A. Singh V. K. Hunter R. L. Jagannath C. (2019). Macrophage Heterogeneity and Plasticity in Tuberculosis. J. Leukoc. Biol. 106 (2), 275–282. doi: 10.1002/JLB.MR0318-095RR 30938876

[B20] Knaul J. K. Jörg S. Oberbeck-Mueller D. Heinemann E. Scheuermann L. Brinkmann V.. (2014). Lung-Residing Myeloid-Derived Suppressors Display Dual Functionality in Murine Pulmonary Tuberculosis. Am. J. Respir. Crit. Care Med. 190 (9), 1053–1066. doi: 10.1164/rccm.201405-0828OC 25275852

[B21] Kotzé L. A. Young C. Leukes V. N. John V. Fang Z. Walzl G.. (2020). Mycobacterium Tuberculosis and Myeloid-Derived Suppressor Cells: Insights Into Caveolin Rich Lipid Rafts. EBioMedicine 53, 102670. doi: 10.1016/j.ebiom.2020.102670 32113158 PMC7047144

[B22] Lee J.-M. Seo J.-H. Kim Y.-J. Kim Y.-S. Ko H.-J. Kang C.-Y. (2012). The Restoration of Myeloid-Derived Suppressor Cells as Functional Antigen-Presenting Cells by NKT Cell Help and All-Trans-Retinoic Acid Treatment. Int. J. Cancer 131 (3), 741–751. doi: 10.1002/ijc.26411 21898392

[B23] Magcwebeba T. Dorhoi A. du Plessis N. (2019). The Emerging Role of Myeloid-Derived Suppressor Cells in Tuberculosis. Front. Immunol. 10. doi: 10.3389/fimmu.2019.00917/full PMC6502992 31114578

[B24] Martino A. Badell E. Abadie V. Balloy V. Chignard M. Mistou M.-Y.. (2010). Mycobacterium Bovis Bacillus Calmette-Guérin Vaccination Mobilizes Innate Myeloid-Derived Suppressor Cells Restraining *In Vivo* T Cell Priming *via* IL-1R-Dependent Nitric Oxide Production. J. Immunol. 15 184 (4), 2038–2047. doi: 10.4049/jimmunol.0903348 20083674

[B25] Pasquinelli V. Quiroga M. F. Martínez G. J. Zorrilla L. C. Musella R. M. Bracco M. M.. (2004). Expression of Signaling Lymphocytic Activation Molecule- Associated Protein Interrupts IFN-γ Production in Human Tuberculosis. J. Immunol. 172 (2), 1177–1185. doi: 10.4049/jimmunol.172.2.1177 14707094

[B26] Pellegrini J. M. Sabbione F. Morelli M. P. Tateosian N. L. Castello F. A. Amiano N. O.. (2021). Neutrophil Autophagy During Human Active Tuberculosis Is Modulated by SLAMF1. Autophagy 17 (9), 2629–2638. doi: 10.1080/15548627.2020.1825273 32954947 PMC8496709

[B27] Rovetta A. I. Peña D. Hernández Del Pino R. E. Recalde G. M. Pellegrini J. Bigi F.. (2014). IFNG-Mediated Immune Responses Enhance Autophagy Against Mycobacterium Tuberculosis Antigens in Patients With Active Tuberculosis. Autophagy 10 (12), 2109–2121. doi: 10.4161/15548627.2014.981791 25426782 PMC4502660

[B28] Salgame P. (2005). Host Innate and Th1 Responses and the Bacterial Factors That Control Mycobacterium Tuberculosis Infection. Curr. Opin. Immunol. 17 (4), 374–380. doi: 10.1016/j.coi.2005.06.006 15963709

[B29] Sampath P. Moideen K. Ranganathan U. D. Bethunaickan R. (2018). Monocyte Subsets: Phenotypes and Function in Tuberculosis Infection. Front. Immunol. 9. doi: 10.3389/fimmu.2018.01726/full PMC6077267 30105020

[B30] Sánchez M. D. García Y. Montes C. París S. C. Rojas M. Barrera L. F.. (2006). Functional and Phenotypic Changes in Monocytes From Patients With Tuberculosis Are Reversed With Treatment. Microbes Infect. 8 (9–10), 2492–2500. doi: 10.1016/j.micinf.2006.06.005 16872859

[B31] Scriba T. J. Kalsdorf B. Abrahams D.-A. Isaacs F. Hofmeister J. Black G.. (2008). Distinct, Specific IL-17- and IL-22-Producing CD4+ T Cell Subsets Contribute to the Human Anti-Mycobacterial Immune Response. J. Immunol. Baltim Md 1950 180 (3), 1962–1970. doi: 10.4049/jimmunol.180.3.1962 PMC2219462 18209095

[B32] Serbina N. V. Lazarevic V. Flynn J. L. (2001). CD4(+) T Cells Are Required for the Development of Cytotoxic CD8(+) T Cells During Mycobacterium Tuberculosis Infection. J. Immunol. 167 (12), 6991–7000. doi: 10.4049/jimmunol.167.12.6991 11739519

[B33] Tateosian N. L. Pellegrini J. M. Amiano N. O. Rolandelli A. Casco N. Palmero D. J.. (2017). IL17A Augments Autophagy in Mycobacterium Tuberculosis-Infected Monocytes From Patients With Active Tuberculosis in Association With the Severity of the Disease. Autophagy 13 (7), 1191–1204. doi: 10.1080/15548627.2017.1320636 28581888 PMC5529075

[B34] Tsiganov E. N. Verbina E. M. Radaeva T. V. Sosunov V. V. Kosmiadi G. A. Nikitina I. Y.. (2014). Gr-1dimcd11b+ Immature Myeloid-Derived Suppressor Cells But Not Neutrophils Are Markers of Lethal Tuberculosis Infection in Mice. J. Immunol. Baltim Md 1950 192 (10), 4718–4727. doi: 10.4049/jimmunol.1301365 PMC4537794 24711621

[B35] World Health Organization Stop TB Initiative (World Health Organization) (2010). Treatment of Tuberculosis: Guidelines. 4th Vol. 147 (Geneva: World Health Organization).

[B36] World Health Organization (2021). Global Tuberculosis Report. Available at: https://www.who.int/teams/global-tuberculosis-programme/tb-reports/global-tuberculosis-report-2021 (Accessed cited 2021 Dec 15).

[B37] Zhan X. Hu S. Wu Y. Li M. Liu T. Ming S.. (2018). IFN-γ Decreased the Suppressive Function of CD33+HLA-DRlow Myeloid Cells Through Down-Regulation of PD-1/PD-L2 Signaling Pathway. Mol. Immunol. 94, 107–120. doi: 10.1016/j.molimm.2017.10.009 29291452

[B38] Ziegler-Heitbrock L. Ancuta P. Crowe S. Dalod M. Grau V. Hart D. N.. (2010). Nomenclature of Monocytes and Dendritic Cells in Blood. Blood 116 (16), e74–e80. doi: 10.1182/blood-2010-02-258558 20628149

