# Supplementary figures and images for: Circulating Monocyte-Like Myeloid Derived Suppressor Cells and CD16 Positive Monocytes Correlate With Immunological Responsiveness of Tuberculosis Patients
Source: Front Cell Infect Microbiol. 2022 Mar 14;12:841741. doi: 10.3389/fcimb.2022.841741 (PMC8964076; doi:10.3389/fcimb.2022.841741)

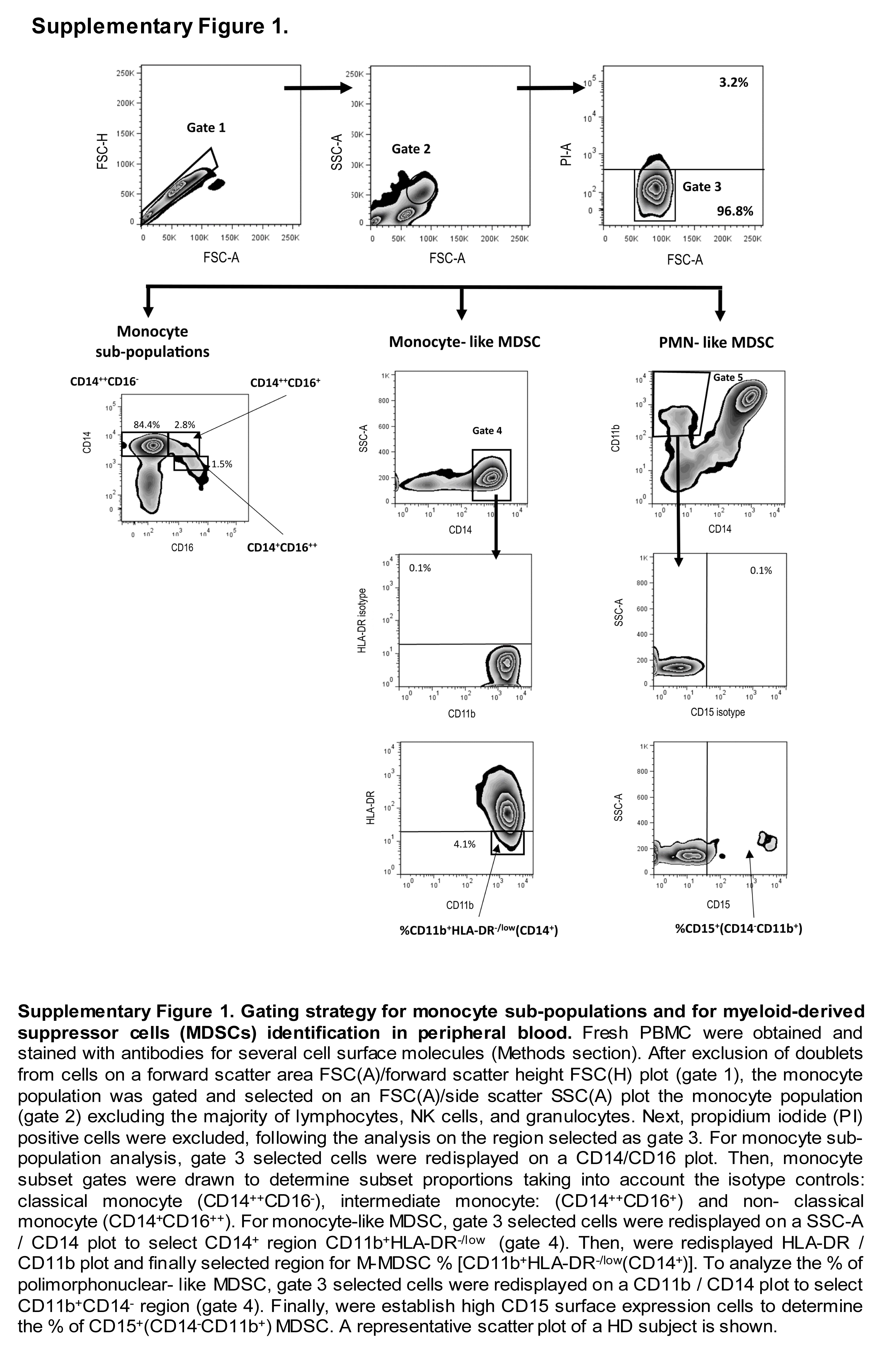

Supplement: Supplementary file 1 [file Image_1.jpg]

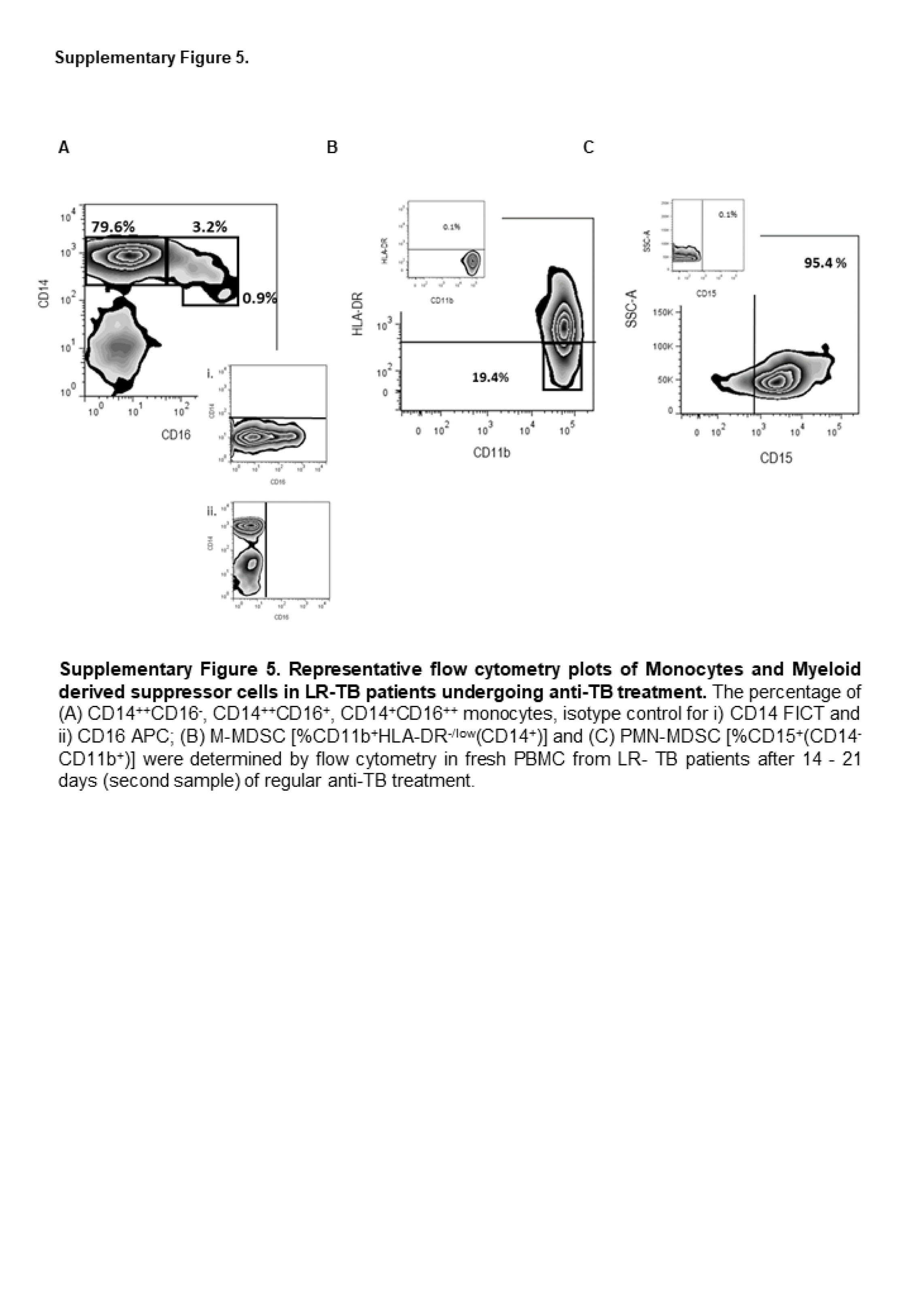

Supplement: Supplementary file 5 [file Image_5.jpg]
